# Supplementary material for: The long shadow of 9/11: Mental health outcomes in adult children of World Trade Center Responders with PTSD
Source: PLOS Ment Health. 2026 May 27;3(5):e0000574. doi: 10.1371/journal.pmen.0000574 (PMC13215529; doi:10.1371/journal.pmen.0000574)
Supplement: S1 Table — (PDF) [file pmen.0000574.s001.pdf]

S1 Table : Association between WTC-R exposure and current parental mental health measures with now-adult children's current mental health, adjusted for child sex, age, race/ethnicity, and parents' sex. Each parent's 9/11 exposure or mental health factor was tested separately. This is presented in Figures 2 and 3.

Factors	*Depression*			*Anxiety*			*Panic*			*PTSD*			*AUD*			*Covid PCL*		
	OR	*95% CI*	*p*	*OR*	*95% CI*	*p*	*OR*	*95% CI*	*p*	*OR*	*95% CI*	*p*	*OR*	*95% CI*	*p*	*Beta*	*95% CI*	*p*
WTC-pR vs. WTC-wR	0.61	[0.29,1.29]	0.19	1.11	[0.58,2.14]	0.74	1.00	[0.52,1.92]	0.99	1.36	[0.54,3.43]	0.51	1.31	[0.65,2.64]	0.44	0.49	[-0.61,1.59]	0.38
Parent's 9/11 exposure																		
Arrival to site	0.53	[0.25,1.14]	0.10	0.60	[0.34,1.07]	***0.08***	0.83	[0.52,1.33]	0.44	0.18	[0.03,0.99]	**0.05**	1.27	[0.62,2.63]	0.51	-0.56	[-1.41,0.29]	0.20
Dust Exposure	0.95	[0.64,1.40]	0.78	0.87	[0.62,1.23]	0.44	0.99	[0.70,1.40]	0.96	0.97	[0.59,1.60]	0.90	1.01	[0.65,1.57]	0.96	-0.43	[-0.98,0.11]	0.12
Worked on the pile	0.84	[0.43,1.64]	0.61	1.28	[0.67,2.46]	0.45	1.80	[0.93,3.49]	***0.08***	0.71	[0.29,1.73]	0.46	1.79	[0.87,3.67]	0.11	0.33	[-0.73,1.39]	0.54
Exposed to remains	1.34	[0.65,2.78]	0.43	1.34	[0.69,2.59]	0.38	1.44	[0.78,2.65]	0.25	4.05	[1.32,12.39]	**0.01**	1.73	[0.78,3.84]	0.18	0.35	[-0.76,1.45]	0.54
South of canal St.	1.07	[0.55,2.10]	0.84	0.99	[0.53,1.84]	0.98	1.23	[0.69,2.19]	0.49	0.84	[0.36,1.98]	0.70	0.75	[0.36,1.54]	0.43	-0.36	[-1.37,0.66]	0.49
Exposure Level	0.85	[0.51,1.43]	0.55	0.95	[0.61,1.49]	0.83	1.17	[0.80,1.70]	0.42	1.08	[0.58,1.99]	0.81	1.54	[0.97,2.43]	***0.07***	-0.06	[-0.68,0.56]	0.85
Total Months on site	0.94	[0.86,1.03]	0.21	0.99	[0.91,1.08]	0.86	1.02	[0.94,1.11]	0.63	1.05	[0.93,1.18]	0.41	1.02	[0.92,1.12]	0.73	0.05	[-0.08,0.19]	0.46
Hours on days 1-2	1.02	[0.99,1.04]	0.23	1.01	[0.99,1.04]	0.22	1.01	[0.98,1.03]	0.56	1.04	[1.01,1.07]	**0.02**	0.99	[0.97,1.02]	0.48	-0.01	[-0.05,0.02]	0.53
Hours on days 3-7	1.01	[1.00,1.02]	0.17	1.01	[1.00,1.02]	**0.02**	1.01	[1.00,1.02]	0.20	1.02	[1.01,1.03]	**0.00**	1.00	[0.99,1.02]	0.51	0.00	[-0.02,0.01]	0.76
Hours on days 8-20	1.00	[1.00,1.01]	0.38	1.00	[1.00,1.01]	**0.01**	1.00	[1.00,1.01]	**0.02**	1.01	[1.00,1.01]	**0.03**	1.00	[1.00,1.01]	0.62	0.00	[0.00,0.01]	0.27
Parent's mental health at interview																		
PTSD	1.61	[1.04,2.51]	**0.03**	1.18	[0.77,1.80]	0.46	1.48	[0.99,2.20]	**0.05**	2.53	[1.33,4.82]	**.005**	1.01	[0.64,1.58]	0.98	0.34	[-0.42,1.10]	0.39
AUD	0.81	[0.28,2.35]	0.70	1.14	[0.45,2.90]	0.78	2.16	[0.82,5.66]	0.12	1.45	[0.31,6.72]	0.63	2.01	[0.76,5.33]	0.16	1.14	[-0.92,3.20]	0.28
Anxiety	1.96	[0.95,4.03]	***0.07***	1.16	[0.57,2.37]	0.68	1.76	[0.94,3.30]	***0.08***	1.17	[0.42,3.28]	0.77	0.84	[0.35,1.99]	0.69	0.59	[-0.63,1.80]	0.35
Depression	2.52	[1.29,4.95]	**0.01**	1.28	[0.69,2.37]	0.43	1.50	[0.83,2.72]	0.18	2.06	[0.86,4.91]	0.10	1.23	[0.59,2.57]	0.59	0.15	[-0.95,1.25]	0.79
Panic	1.48	[0.78,2.82]	0.23	0.92	[0.49,1.71]	0.79	1.22	[0.64,2.33]	0.54	2.87	[1.21,6.78]	**0.02**	0.84	[0.42,1.70]	0.63	0.19	[-0.97,1.35]	0.75
Covid PCL	1.00	[0.89,1.12]	1.00	0.95	[0.85,1.06]	0.33	1.02	[0.93,1.12]	0.67	1.01	[0.87,1.18]	0.85	1.07	[0.97,1.18]	0.19	0.09	[-0.07,0.24]	0.27
Parent's Other Factors																		
Cancer	1.48	[0.68,3.26]	0.33	1.76	[0.88,3.51]	0.11	1.10	[0.56,2.16]	0.78	1.14	[0.41,3.19]	0.80	1.58	[0.79,3.19]	0.20	0.79	[-0.35,1.93]	0.17
Life Events	1.11	[1.03,1.20]	**0.01**	1.07	[1.00,1.14]	***0.06***	1.03	[0.96,1.11]	0.42	1.07	[0.97,1.19]	0.19	1.05	[0.97,1.14]	0.25	-0.02	[-0.14,0.09]	0.70

Note: OR - odds ratios, LCL - lower confidence level, UCL- upper confidence level; Bold fonts represent $p<0.05$, and Italic bold fonts represent $0.05 < p < 0.1$.
