# Supplementary material for: The long shadow of 9/11: Mental health outcomes in adult children of World Trade Center Responders with PTSD
Source: PLOS Ment Health. 2026 May 27;3(5):e0000574. doi: 10.1371/journal.pmen.0000574 (PMC13215529; doi:10.1371/journal.pmen.0000574)
Supplement: S2 Table — (PDF) [file pmen.0000574.s002.pdf]

S2 Table: Weighted model of the Association between WTC-R exposure and current parental mental health measures with now-adult children's current mental health, adjusted for child sex, age, race/ethnicity, and parents' sex. Each parental 9/11 exposure or mental health factor is tested separately. This model is similar to the unweighted model presented in Table 6AS above.

Factors	*Depression*			*Anxiety*			*Panic*			*PTSD*			*AUD*			*Covid PCL*		
	OR	*95% CI*	*p*	*OR*	*95% CI*	*p*	*OR*	*95% CI*	*p*	*OR*	*95% CI*	*p*	*OR*	*95% CI*	*p*	*Beta*	*95% CI*	*p*
WTC-pR vs. WTC-wR	0.66	[0.30,1.44]	0.30	1.33	[0.67,2.62]	0.42	1.37	[0.69,2.70]	0.37	1.10	[0.55,2.19]	0.79	1.53	[0.61,3.87]	0.37	0.56	[-0.50,1.62]	0.30
Parent's 9/11 exposure																		
Arrival to site	0.51	[0.23,1.15]	0.10	0.62	[0.33,1.16]	0.13	1.10	[0.54,2.26]	0.79	0.86	[0.52,1.43]	0.57	0.17	[0.03,0.95]	**0.04**	-0.42	[-1.26,0.43]	0.33
Dust Exposure	0.89	[0.58,1.37]	0.61	0.90	[0.61,1.32]	0.58	1.01	[0.64,1.59]	0.96	1.03	[0.71,1.50]	0.88	0.96	[0.55,1.67]	0.88	-0.35	[-0.91,0.22]	0.23
Worked on the pile	1.13	[0.53,2.42]	0.76	1.82	[0.88,3.75]	0.11	2.22	[1.07,4.60]	**0.03**	2.26	[1.13,4.54]	**0.02**	1.08	[0.42,2.81]	0.87	0.61	[-0.44,1.66]	0.26
Exposed to remains	1.35	[0.61,3.00]	0.46	1.45	[0.71,2.99]	0.31	1.86	[0.79,4.36]	0.15	1.58	[0.82,3.02]	0.17	5.20	[1.51,17.84]	**0.01**	0.34	[-0.77,1.46]	0.55
South of canal St.	0.93	[0.45,1.92]	0.84	0.99	[0.50,1.94]	0.97	0.76	[0.37,1.60]	0.48	1.11	[0.60,2.07]	0.73	0.80	[0.32,2.00]	0.63	-0.42	[-1.47,0.63]	0.43
Exposure Level	0.88	[0.50,1.55]	0.67	1.05	[0.66,1.68]	0.84	1.46	[0.91,2.32]	0.11	1.29	[0.86,1.92]	0.21	1.17	[0.62,2.23]	0.63	0.03	[-0.56,0.63]	0.91
Total Months on site	0.96	[0.87,1.07]	0.51	1.01	[0.92,1.11]	0.85	1.00	[0.90,1.11]	0.95	1.04	[0.95,1.14]	0.43	1.08	[0.95,1.23]	0.24	0.07	[-0.07,0.21]	0.30
Hours on days 1-2	1.01	[0.99,1.04]	0.39	1.01	[0.99,1.04]	0.27	0.99	[0.97,1.02]	0.61	1.01	[0.98,1.03]	0.63	1.04	[1.00,1.07]	**0.03**	-0.01	[-0.04,0.02]	0.54
Hours on days 3-7	1.01	[1.00,1.02]	0.16	1.01	[1.00,1.02]	**0.02**	1.00	[0.99,1.02]	0.65	1.01	[1.00,1.02]	0.13	1.02	[1.01,1.04]	**0.00**	0.00	[-0.02,0.01]	0.85
Hours on days 8-20	1.00	[1.00,1.01]	0.34	1.01	[1.00,1.01]	**0.01**	1.00	[1.00,1.01]	0.72	1.01	[1.00,1.01]	**0.01**	1.01	[1.00,1.01]	**0.02**	0.00	[0.00,0.01]	0.29
Parent's mental health at interview																		
PTSD	1.56	[0.97,2.50]	**0.07**	1.09	[0.70,1.71]	0.70	1.01	[0.64,1.61]	0.95	1.35	[0.89,2.05]	0.15	2.13	[1.10,4.12]	**0.02**	0.12	[-0.59,0.84]	0.74
AUD	0.71	[0.23,2.20]	0.55	0.92	[0.34,2.47]	0.86	1.68	[0.61,4.59]	0.31	1.77	[0.67,4.66]	0.25	1.02	[0.21,4.94]	0.99	0.86	[-1.08,2.81]	0.38
Anxiety	1.99	[0.92,4.29]	***0.08***	1.12	[0.53,2.37]	0.76	0.81	[0.34,1.97]	0.65	1.64	[0.84,3.17]	0.14	0.85	[0.30,2.44]	0.77	0.47	[-0.70,1.64]	0.43
Depression	2.12	[1.02,4.44]	**0.05**	1.10	[0.56,2.15]	0.79	1.01	[0.48,2.15]	0.98	1.34	[0.72,2.49]	0.35	1.56	[0.63,3.87]	0.34	0.05	[-1.01,1.11]	0.93
Panic	1.37	[0.68,2.77]	0.38	0.76	[0.39,1.49]	0.43	0.75	[0.36,1.55]	0.44	1.08	[0.56,2.06]	0.82	2.19	[0.88,5.44]	0.09	-0.05	[-1.14,1.03]	0.92
Covid PCL	0.99	[0.87,1.13]	0.85	0.93	[0.82,1.06]	0.30	1.06	[0.97,1.17]	0.20	1.00	[0.90,1.11]	0.95	1.02	[0.87,1.20]	0.81	0.07	[-0.09,0.22]	0.41
Parent's Other Factors																		
Cancer	1.52	[0.66,3.49]	0.32	1.64	[0.78,3.46]	0.19	1.72	[0.85,3.50]	0.13	1.09	[0.53,2.23]	0.82	1.15	[0.41,3.21]	0.79	0.52	[-0.60,1.65]	0.36
Life Events	1.11	[1.02,1.21]	**0.01**	1.07	[0.99,1.15]	0.09	1.05	[0.96,1.15]	0.29	1.03	[0.96,1.11]	0.46	1.06	[0.95,1.18]	0.33	-0.02	[-0.13,0.09]	0.75

Note: OR - odds ratios, LCL - lower confidence level, UCL- upper confidence level; Bold fonts represent $p<0.05$, and Italic bold fonts represent $0.05 < p < 0.1$.
