# Supplementary material for: The long shadow of 9/11: Mental health outcomes in adult children of World Trade Center Responders with PTSD
Source: PLOS Ment Health. 2026 May 27;3(5):e0000574. doi: 10.1371/journal.pmen.0000574 (PMC13215529; doi:10.1371/journal.pmen.0000574)
Supplement: S3 Table — (PDF) [file pmen.0000574.s003.pdf]

S3 Table : Association between WTC-R Social Support measures and current mental health of the now-adult children, adjusted for child sex, age, race/ethnicity, and parents' sex and occupational category. Each parent's 9/11 exposure or mental health factor was tested separately.

Factors	***Depression***			***Anxiety***			***Panic***			***PTSD***			***AUD***			***Covid PCL***		
	OR	***95% CI***	***p***	***OR***	***95% CI***	***p***	***OR***	***95% CI***	***p***	***OR***	***95% CI***	***p***	***OR***	***95% CI***	***p***	***Beta***	***95% CI***	***p***
Parent's Social Support																		
PARQ- Negative sub	1.17	[1.03,1.33]	**0.02**	1.10	[0.97,1.25]	0.13	1.11	[0.98,1.26]	***0.09***	1.15	[0.96,1.39]	0.13	1.15	[1.02,1.31]	**0.03**	0.01	[-0.16,0.19]	0.89
PARQ - Positive sub	0.94	[0.85,1.05]	0.27	0.90	[0.82,0.99]	**0.02**	1.00	[0.91,1.11]	0.94	0.94	[0.82,1.07]	0.32	0.93	[0.84,1.02]	0.14	0.00	[-0.16,0.16]	1.00
General Social Support	0.92	[0.83,1.02]	0.13	0.99	[0.89,1.09]	0.82	0.97	[0.88,1.07]	0.55	0.84	[0.73,0.96]	**0.01**	0.92	[0.82,1.02]	0.12	-0.08	[-0.23,0.06]	0.27
Spouse/partner Support	2.17	[1.21,3.89]	**0.01**	1.61	[0.94,2.73]	***0.08***	1.11	[0.62,1.98]	0.74	2.37	[1.06,5.31]	**0.04**	1.60	[0.73,3.54]	0.24	-0.16	[-1.23,0.90]	0.76
Spouse/partner strain	0.59	[0.32,1.08]	*0.09*	0.88	[0.54,1.46]	0.63	0.78	[0.47,1.28]	0.32	0.60	[0.26,1.38]	0.23	1.05	[0.56,1.98]	0.87	-0.16	[-1.02,0.71]	0.72
Family support	1.33	[0.78,2.28]	0.29	1.02	[0.61,1.69]	0.95	0.74	[0.46,1.17]	0.20	1.07	[0.55,2.09]	0.85	1.35	[0.76,2.40]	0.31	-0.41	[-1.15,0.33]	0.28
Family strain	0.54	[0.34,0.87]	**0.01**	0.97	[0.62,1.51]	0.88	1.05	[0.68,1.62]	0.83	0.66	[0.34,1.29]	0.22	0.45	[0.26,0.77]	**0.00**	0.33	[-0.47,1.13]	0.42
Friend support	1.10	[0.71,1.72]	0.66	1.18	[0.80,1.74]	0.41	1.00	[0.66,1.53]	0.98	1.72	[0.95,3.13]	***0.07***	1.63	[0.98,2.71]	***0.06***	0.62	[-0.15,1.39]	0.12
Friend strain	0.61	[0.35,1.06]	***0.08***	1.00	[0.60,1.65]	0.99	1.31	[0.76,2.25]	0.33	0.61	[0.26,1.41]	0.25	0.58	[0.31,1.06]	***0.08***	0.36	[-0.55,1.27]	0.44
Resilience	0.88	[0.72,1.06]	0.18	0.88	[0.74,1.04]	0.12	1.02	[0.84,1.24]	0.82	0.93	[0.73,1.17]	0.52	0.90	[0.75,1.09]	0.30	-0.16	[-0.48,0.15]	0.31
Quality of life	0.96	[0.93,1.00]	**0.03**	1.00	[0.97,1.03]	0.81	0.98	[0.95,1.01]	0.24	0.95	[0.90,1.00]	***0.07***	0.99	[0.96,1.03]	0.71	-0.03	[-0.09,0.02]	0.22

Note: PARQ scoring higher negative PARQ scores and lower positive PARQ scores indicate poorer parent-child relationship. Perceived Support scoring: Higher support scores indicate lower perceived support, and higher strain scores indicate lower perceived support.
OR - odds ratios, LCL - lower confidence level, UCL- upper confidence level
Bold fonts represent $p<0.05$, and Italic bold fonts represent $0.05 < p < 0.1$.
