# Supplementary material for: The long shadow of 9/11: Mental health outcomes in adult children of World Trade Center Responders with PTSD
Source: PLOS Ment Health. 2026 May 27;3(5):e0000574. doi: 10.1371/journal.pmen.0000574 (PMC13215529; doi:10.1371/journal.pmen.0000574)
Supplement: S1 Text — (PDF) [file pmen.0000574.s004.pdf]

S1 Text: General Social Support (3 items from the MOS Social Support Survey)

How often is each of the following kinds of support available to you if you need it?

Someone to love you and make you feel wanted
Someone to help you if you were confined to bed
Someone to give you good advice in a crisis

Scale:

0	None of the time
1	A little of the time
2	Some of the time
3	Most of the time
4	All of the time
