# Supplementary material for: The long shadow of 9/11: Mental health outcomes in adult children of World Trade Center Responders with PTSD
Source: PLOS Ment Health. 2026 May 27;3(5):e0000574. doi: 10.1371/journal.pmen.0000574 (PMC13215529; doi:10.1371/journal.pmen.0000574)
Supplement: S2 Text — (PDF) [file pmen.0000574.s005.pdf]

S2 Text: COVID Questionnaire

The COVID instrument was developed by our research team during the early stages of the COVID-19 pandemic in 2020 and, therefore, included items reflecting concerns that were most relevant at that time.

1. COVID questions about self

Now we will ask about your experiences during the COVID-19 pandemic.
Did you have Coronavirus infection?<br>0 No<br>1 Yes, had symptoms but no diagnosis by doctor<br>2 Yes, medical diagnosis but no test<br>3 Yes, had a positive test
Were you seriously ill from COVID-19?<br>0 No<br>1 Yes
Were you hospitalized from COVID-19?<br>0 No<br>1 Yes

2. COVID about people living in same household

Did anyone living with you within the last year have Coronavirus infection?<br>0 No<br>1 Yes
Did anyone living with you within the last year get seriously ill from COVID-19?<br>0 No<br>1 Yes
Who was seriously ill from COVID-19? (PLEASE SELECT ALL THAT APPLY)<br>1 spouse/partner<br>2 mother<br>3 father<br>4 child1<br>5 child2<br>6 child3<br>7 child4<br>8 child5

9	child6
10	sister1
11	sister2
12	sister3
13	brother1
14	brother2
15	brother3
16	roommate 1
17	roommate 2
18	roommate 3
Did anyone living with you within the last year get hospitalized from COVID-19?	
0	No
1	Yes
Who was hospitalized from COVID-19? -*Same choices as before (1-18)*	
Did anyone living with you within the last year pass away from COVID-19?	
0	No
1	Yes
Who passed away from COVID-19?- *Same choices as before (1-18)*	

3. COVID about Family living in a different household

Did anyone in your family, who was not living with you during the last year, have Coronavirus infection?	
0	No
1	Yes
Did anyone in your family, who was not living with you during the last year, get seriously ill from COVID-19?	
0	No
1	Yes
Who was seriously ill from COVID-19?<br><br>(PLEASE SELECT ALL THAT APPLY)- *Same choices as before (1-18)*	
Did anyone in your family, who was not living with you during the last year, get hospitalized from COVID-19?	
0	No
1	Yes
Who was hospitalized from COVID-19?<br><br>(PLEASE SELECT ALL THAT APPLY)- *Same choices as before (1-18)*	

Did anyone in your family, who was not living with you during the last year, pass away from COVID-19?
Who passed away from COVID-19?<br><br>(PLEASE SELECT ALL THAT APPLY)- *Same choices as before (1-18)*

4. COVID about Close Friends

How many of your close friends got coronavirus infection?
How many of your close friends were seriously ill from COVID-19?
How many of your close friends were hospitalized from COVID-19?
How many of your close friends passed away from COVID-19?

5. COVID worries

Are you worried you will get sick (again) with Coronavirus?<br>1 Extremely worried<br>2 Very worried<br>3 Somewhat worried<br>4 A little worried<br>5 Not at all worried
Are you worried your adult-children may get sick with Coronavirus?<br>1 Extremely worried<br>2 Very worried<br>3 Somewhat worried<br>4 A little worried<br>5 Not at all worried
Are you worried your parent(s) may get sick with Coronavirus?<br>1 Extremely worried<br>2 Very worried<br>3 Somewhat worried<br>4 A little worried<br>5 Not at all worried

6. COVID Job loss /living situation

As a result of the COVID-19 pandemic, have you...<br>(PLEASE SELECT ALL THAT APPLY)<br>1 Lost your job<br>2 Changed living conditions (e.g. move out of apt)<br>0 None of the above

As a result of the COVID-19 pandemic, has anyone in your family, or have any of your close friends ...<br>(PLEASE SELECT ALL THAT APPLY)<br>1 Lost their job<br>2 Changed living conditions (e.g. move out of apt)<br>0 None of the above
Who lost their job from COVID-19?<br><br>(PLEASE SELECT ALL THAT APPLY)<br>1 mother<br>2 father<br>3 spouse<br>4 son<br>5 daughter<br>6 brother<br>7 sister<br>8 close friend<br>10 roommate<br>9 other
Please describe "Other"
Who changed their living situation from COVID-19?<br><br>(PLEASE SELECT ALL THAT APPLY)<br>1 mother<br>2 father<br>3 spouse<br>4 son<br>5 daughter<br>6 brother<br>7 sister<br>8 close friend<br>10 roommate<br>9 other
**7.** Please describe "Other"
Were you a frontline worker during the COVID-19 pandemic?<br>1 Doctor<br>2 Nurse<br>3 Driver<br>4 Other<br>0 No, not a frontline worker
Please describe "Other":

8. COVID short-PCL – questions 1,7,9, 18 of PCL with the following scale: 0 - Not at all ; 1 - A little bit; 2 – Moderately; 3 - Quite a bit; 4 – Extremely.

In the past month, how much were you bothered by the following?
Repeated, disturbing, and unwanted memories of the COVID-19 pandemic?
Avoiding external reminders of your experiences related to the COVID-19 pandemic (for example, people, places, conversations, activities, objects, or situations)?
Having strong negative beliefs about yourself, other people, or the world because of the COVID-19 pandemic (for example, having thoughts such as: I am bad, there is something seriously wrong with me, no one can be trusted, the world is completely dangerous)?
Feeling jumpy or easily startled because of the COVID-19 pandemic?

The Long Shadow of 9/11: Mental Health Outcomes in Adult Children of World Trade Center Responders with PTSD
