# Supplementary material for: The long shadow of 9/11: Mental health outcomes in adult children of World Trade Center Responders with PTSD
Source: PLOS Ment Health. 2026 May 27;3(5):e0000574. doi: 10.1371/journal.pmen.0000574 (PMC13215529; doi:10.1371/journal.pmen.0000574)
Supplement: S1 Appendix — (PDF) [file pmen.0000574.s006.pdf]

S1 Appendix. Tables A through E include data for the overall sample, which are not reported in Tables 2 through 5 in the main paper.

<u>Table A:</u> Demographic characteristics of the WTC-R parents overall, and separately for WTC-wR and WTC-pR groups.

	**Total (N=176)**		**WTC-wR (N=93)**		**WTC-pR (N=81)**			
	**Mean**	**SD**	**Mean**	**SD**	**Mean**	**SD**	**p**	missing
Age (years)	59.82	5.72	60.74	5.72	58.81	5.61	***0.026***	
	**N**	**%**	**N**	**%**	**N**	**%**		
Sex							***0.001***	
Female	24	13.64	3	3.23	21	25.93		
Male	152	86.36	90	96.77	60	74.07		
Ethnicity Spanish speaking	32	18.39	17	18.48	14	17.50	0.868	2
Race and ethnicity							0.560	11
Black, non-Hispanic	7	4.24	2	2.30	5	6.58		
Black, Hispanic	7	4.24	4	4.60	2	2.63		
White, non-Hispanic	116	70.30	61	70.12	54	71.05		
White, Hispanic	20	12.12	10	11.49	10	13.16		
Other groups	11	6.67	8	9.20	3	3.95		
More than one group	4	2.42	2	2.30	2	2.63		
Education							***0.013***	2
HS or less	27	15.52	22	23.91	5	6.25		
Some college/Assoc. degree	86	49.43	43	46.74	42	52.50		
Bachelors degree	40	22.99	19	20.65	21	26.25		
Graduate degree	21	12.07	8	8.70	12	15.00		
Employed (full or part time)	65	37.57	35	38.89	28	34.57	0.559	3
Income							***0.028***	3
Up to $99,000	58	33.53	40	43.96	18	22.50		
$100,000-199,999	73	42.20	34	37.36	38	47.50		
$200,000 or above	25	14.45	10	10.99	14	17.50		
Prefer not to answer	17	9.83	7	7.69	10	12.50		
Married	129	73.71	64	68.82	63	78.75	0.140	1
Lives with a partner	127	72.16	58	62.37	67	82.72	***0.003***	
Lives with child(ren)	88	50.00	42	45.16	44	54.32	0.228	

<u>Table B:</u> Demographic characteristics of the children of the WTC-R, overall, and separately based on their parents' occupational roles as WTC-wR or WTC-pR.

	**Total (N=270)**		**WTC-wR (N=134)**		**WTC-pR (N=119)**			
	**Mean**	**SD**	**Mean**	**SD**	**Mean**	**SD**	**p**	**missing**
Age (years)	28.69	5.11	28.69	5.29	28.66	4.92	0.952	
	**N**	**%**	**N**	**%**	**N**	**%**		
Sex							0.747	6
Female	166	61.70	83	63.85	73	61.86		
Male	98	36.40	47	36.15	45	38.14		
Ethnicity Spanish speaking	78	29.55	37	28.68	36	30.25	0.786	6
Race and ethnicity							0.548	9
Black, non-Hispanic	7	2.68	2	1.56	5	4.24		
Black, Hispanic	6	2.30	2	1.56	2	1.70		
White, non-Hispanic	168	64.37	82	64.06	76	64.41		
White, Hispanic	41	15.71	18	14.06	21	17.80		
Other groups	7	2.68	4	3.13	2	1.70		
More than one group	32	12.26	20	15.63	12	10.17		
Education							0.147	3
HS or less	23	8.61	12	9.09	8	6.78		
Some college/Assoc. degree	72	26.97	39	29.55	26	22.03		
Bachelors degree	113	42.32	47	35.61	59	50.00		
Graduate degree	59	22.10	34	25.76	25	21.19		
Employed (full or part time)	214	81.06	107	82.31	95	81.20	0.821	6
Income							0.406	7
Up to $99,000	129	49.05	70	53.85	51	43.97		
$100,000-199,999	71	27.00	32	24.62	37	31.90		
$200,000 or above	19	7.22	9	6.92	7	6.03		
Prefer not to answer	44	16.73	19	14.62	21	18.10		
Married	68	25.19	35	26.12	28	23.53	0.634	
Lives with a partner	105	38.89	50	37.31	49	41.18	0.530	
Lives with parents	103	38.15	50	37.31	47	39.50	0.722	
Lives with child(ren)	52	19.26	27	20.15	23	19.33	0.870	

Table C: Exposure characteristics to the terror attack of the WTC-R parents overall, and separately for WTC-wR and WTC-pR groups.

	**Total (N=176)**		**WTC-wR (N=93)**		**WTC-pR (N=81)**			
	**N**	**%**	**N**	**%**	**N**	**%**	**p**	**missing**
Arrival category							0.076	2
9/11-9/12	136	78.16	67	72.04	69	85.19		
9/13-9/17	29	16.67	21	22.58	8	9.88		
9/18 and later	9	5.17	5	5.38	4	4.94		
Dust exposure							0.099	5
None	69	40.35	44	47.83	25	31.65		
Some, not in cloud	49	28.66	23	25.00	26	32.91		
In cloud	53	30.99	25	27.17	28	35.44		
Ever worked on the pile/in the pit	95	56.21	48	52.17	47	61.04	0.250	7
Was exposed to human remains	98	61.64	44	53.01	54	72.97	***0.010***	17
Was South of Canal St. on 9/11	105	60.69	49	52.69	56	70.00	***0.020***	3
	**Mean**	**SD**	**Mean**	**SD**	**Mean**	**SD**		
Level of exposure (range, 0-3)	1.29	0.73	1.17	0.68	1.42	0.76	***0.030***	8
Total exposure (months)	4.24	3.54	3.93	3.83	4.61	3.16	0.206	7
Hours at the site								
On days 1-2	17.12	12.94	14.58	12.68	20.46	12.46	***0.002***	
On days 3-7	44.38	28.38	43.15	28.29	46.89	28.00	0.383	
On days 8-20	100.52	74.79	96.23	75.09	107.92	73.64	0.302	

<u>Table D:</u> Mental health characteristics of the WTC-R parents and their children overall, and separately stratified by WTC-wR and WTC-pR groups.

	**Total**		**WTC-wR**		**WTC-pR**			
	**Mean**	**SD**	**Mean**	**SD**	**Mean**	**SD**	**p**	**missing**
Life Traumatic Events								
Parents	11.48	4.54	10.22	4.73	12.96	3.88	**<.0001**	
Adult-Children	9.08	4.83	8.69	4.86	9.82	4.63	**0.060**	1
	**N**	**%**	**N**	**%**	**N**	**%**		
**A. Parents**								
Depression	56	31.82	31	33.33	23	28.40	0.480	
Anxiety disorder	43	24.43	26	27.96	15	18.52	0.140	
PTSD (vs. negative)							0.730	26*
Possible PTSD	33	22.00	14	18.92	18	24.32		
PTSD	26	17.33	13	17.57	12	16.22	.	
AUD	15	8.52	8	8.60	7	8.64	0.990	
SUD	1	0.57	.	.	1	1.24	0.280	
Panic								
score > 8.75	54	30.68	28	30.11	25	30.86	0.910	
Not of psychiatric origin	13	7.39	8	8.60	5	6.17	0.540	
**B. Adult-Children**								
Depression	59	21.85	29	21.64	25	21.01	0.902	
Anxiety disorder	68	25.19	30	22.39	34	28.57	0.259	
PTSD (vs. negative)							0.578	101*
Possible PTSD	16	9.47	6	6.98	9	11.54		
PTSD	12	7.10	6	6.98	6	7.69	.	
AUD	46	17.10	21	15.67	24	20.34	0.334	1
SUD	4	1.48	3	2.24	1	0.84	0.373	
Panic								
score > 8.75	74	27.41	38	28.36	32	26.89	0.795	
Not of psychiatric origin	14	5.19	5	3.73	9	7.56	0.183	

Note: * Missing values due to a technical failure during data collection. Possible PTSD was defined as scores between 31 and 43, whereas PTSD was defined as scores ≥44.

Table E: Social support, relationships, quality of life, and resilience scores of the WTC-R parents and their children overall, and separately stratified by WTC-wR and WTC-pR group.

	**Total**		**WTC-wR**		**WTC-pR**			
	**Mean**	**SD**	**Mean**	**SD**	**Mean**	**SD**	**p**	**Missing**
**A. Parents**								
PARQ								
Negative subscale	7.30	2.52	7.39	2.69	7.24	2.32	0.702	2
Positive subscale	16.38	3.21	16.01	3.31	16.78	3.06	0.113	2
General social support	8.85	3.13	8.74	3.34	9.01	2.87	0.568	1
Perceived Social Support								
Spouse/partner support	1.40	0.55	1.36	0.53	1.42	0.54	0.572	33[a]
Spouse/partner strain	2.80	0.68	2.81	0.72	2.79	0.64	0.866	33[a]
Family support	1.65	0.61	1.68	0.64	1.60	0.58	0.393	2
Family strain	2.95	0.69	3.00	0.71	2.89	0.65	0.273	2
Friend support	1.87	0.76	1.92	0.73	1.80	0.77	0.324	3
Friend strain	3.19	0.57	3.20	0.59	3.16	0.54	0.654	3
Resilience	8.40	1.55	8.45	1.43	8.35	1.66	0.670	1
Quality of life	47.69	9.98	47.32	10.80	48.35	8.96	0.495	1
**A. Adult-Children**								
PARQ								
Negative subscale	9.22	3.53	9.45	3.46	9.00	3.49	0.303	7
Positive subscale	16.26	3.24	16.11	3.07	16.55	3.37	0.287	8
General social support	9.80	2.66	9.50	2.79	10.21	2.40	*0.032*	10
Perceived Social Support								
Spouse/partner support	1.23	0.36	1.25	0.39	1.19	0.34	0.361	107[a]
Spouse/partner strain	3.11	0.64	3.11	0.65	3.12	0.62	0.947	107[a]
Family support	1.50	0.47	1.48	0.44	1.50	0.50	0.785	9
Family strain	2.73	0.70	2.71	0.66	2.77	0.71	0.487	9
Friend support	1.57	0.60	1.60	0.59	1.56	0.62	0.573	12
Friend strain	3.17	0.54	3.13	0.53	3.22	0.55	0.199	13
Resilience	6.21	1.45	6.24	1.45	6.14	1.45	0.586	10
Quality of life	51.68	9.35	52.13	9.11	51.31	9.64	0.494	9

Note: PARQ scoring higher negative PARQ scores and lower positive PARQ scores indicate poorer parent-child relationship. Perceived Support scoring: Higher support scores indicate lower perceived support, and higher strain scores indicate lower perceived support. [a]Indicating a marital status of being single.
